# Negative expectations and measurable movement mechanics: a scoping review of the nocebo effect on motor performance

**DOI:** 10.3389/fnhum.2025.1666804

**Published:** 2025-09-24

**Authors:** Jennifer Burgos-Tirado, Guillaume Léonard, Adrien Hakimi, Deborah Vancraeynest, Thierry Lelard, Maryne Cozette

**Affiliations:** ^1^UR-UPJV EA 3300, APERE - Adaptations Physiologiques á l’Exercice et Réadaptation á l’effort - UFR des Sciences du Sport, Université de Picardie Jules Verne, Amiens, France; ^2^Research Centre on Aging, CIUSSS de l’Estrie – CHUS, Sherbrooke, QC, Canada; ^3^School of Rehabilitation, Faculty of Medicine and Health Sciences, Université de Sherbrooke, Sherbrooke, QC, Canada

**Keywords:** pain perception, kinematics, avoidance learning, psychological conditioning, motor control, nocebo effect, psychomotor performance

## Abstract

**Introduction:**

The nocebo effect, where negative responses can occur as a result of negative expectations, has gained increasing attention in motor control research, with growing evidence highlighting its impact on both athletic and everyday movements. However, the specific methodologies used to induce nocebo effects on motor outcomes remain unexplored. This scoping review aimed to address three key questions: (1) What experimental protocols have been developed and used to elicit nocebo effects in motor performance in healthy individuals? (2) How are these effects assessed and measured? (3) What are the observed effects on motor outcomes?

**Methods:**

A scoping review was conducted following the PRISMA framework, searching PubMed, EBSCO, EMBASE, Cochrane, Web of Science, Scopus, and Google Scholar from inception to March 2025. Eighteen studies were included.

**Results and discussion:**

Verbal instruction was the most common induction method (94.4 %), often combined with conditioning or visual cues. Motor tasks assessed gross skills, such as sprinting and cycling), with limited attention given to fine motor control. Outcomes were more frequently centered on performance measures (e.g., strength, endurance), with less emphasis on movement quality (e.g., coordination). Nocebo effects were observed in half of the studies impairing motor performance, including reduced force production, diminished endurance, disrupted postural stability and slower movement speed. The findings highlight methodological diversity in induction protocols and measurement methods. Future research should expand participant diversity, investigate fine motor tasks, and further explore the interplay between induction methods and motor outcomes.

## Introduction

Human experience is shaped by the dynamic interplay between internal states (emotions and expectations) and motricity (which encompasses everything from subtle gestures to complex athletics skills). This mind–body connection affects movement (Mahfoudi et al., 2023; Vernazza-Martin et al., 2022), decision making (El Zein et al., 2024; Tamir et al., 2015), and self-care (Glattacker et al., 2022). Neuroscientific evidence increasingly highlights that brain regions linked to emotion directly influence motor control pathways impacting movement precision and fluidity (Tamir et al., 2015; Lima Portugal et al., 2020; Léonard et al., 2025; Beatty et al., 2016). Similarly, expectations about the consequences of a future event or one’s own abilities can significantly enhance or impair performance (Colloca and Benedetti, 2016; Chavarria et al., 2017), guiding motor planning and even the perception of effort and pain and when these expectations are negative, they can elicit what is known as the “nocebo effect.”

In definition, the nocebo effect refers to negative responses that can occur during medical treatments or clinical trials, even when a placebo or inactive treatment is administered (Chavarria et al., 2017; Colloca and Finniss, 2012; Blasini et al., 2017; Colloca, 2024; Colloca and Barsky, 2020). These responses are not explained by the pharmacological effects of the treatment itself but are related to the patient’s negative expectations (Colloca and Finniss, 2012; Colloca, 2024; Benedetti et al., 2003) or conditioning (Benedetti et al., 2003; Benedetti et al., 2003). These can be triggered by verbal suggestions (Colloca and Finniss, 2012), previous negative experiences (Colloca and Benedetti, 2016; Benedetti et al., 2003), observation of others experiencing adverse outcomes (Klauß et al., 2024), and various contextual and environmental factors (Chavarria et al., 2017; Colloca and Finniss, 2012; Colloca, 2024; Colloca and Miller, 2011; Häuser et al., 2012). Historically, this phenomenon was considered a nuisance in clinical trials, making drug validation difficult due to the occurrence of side effects in placebo groups (Colloca, 2024; Häuser et al., 2012; Planès et al., 2016). However, the nocebo effect is now recognized as a significant psychobiological phenomenon with implications beyond clinical settings, extending into rehabilitation, sports performance, and even daily functioning activities.

Several systematic reviews have investigated placebo and nocebo effects on sports and motor performance (Fiorio, 2018; Hurst et al., 2020; Horváth et al., 2021; Grosso et al., 2024; Chhabra and Szabo, 2024): in a recent review (Hurst et al., 2020) about how negative and positive expectations impair athletic skills, it was suggested that negative expectations may impair athletic performance twice as much as positive enhance it. Another systematic review further confirms the significant impact of nocebo effects on motor performance (Horváth et al., 2021). Negative expectations may lead to unnecessary movement limitations, reduced treatment efficacy, and impaired functional capacity (Horváth et al., 2021; Grosso et al., 2024). Paradoxically, the nocebo phenomenon remains disproportionately understudied. These robust effects carry significant implications across multiple domains - from elite sports (Beedie et al., 2007; Hurst et al., 2017; Hurst et al., 2020) performance to rehabilitation outcomes and activities of daily living (Chavarria et al., 2017; Colloca and Miller, 2011; Nishi et al., 2021).

In the case of sports performance, for example, one study (Beedie et al., 2007) exposed athletes to a fictitious supplement (cornstarch in a gelatin capsule), and found that the negative beliefs about the effects of the supplement lead to reduced running speed compared to baseline performance. Similarly, a pilot study (McLemore et al., 2020) revealed that untrained males who were told an inert cornstarch capsule would increase muscle soreness showed significantly lower range of motion and fewer exercise repetitions compared to controls. These observations suggest how negative expectations alone can significantly hinder physical performance, even in the absence of any active substance.

Nonetheless, the extent to which the nocebo effect manifests in the realm of physical performance remains an area of ongoing inquiry. Beyond these findings, nocebo suggestions have been linked to broader impairments, including diminished muscle strength (Zech et al., 2019), reduced endurance performance (McLemore et al., 2020; De La Vega et al., 2017), increased fatigue perception and effort (Corsi et al., 2019; Horváth et al., 2024), as well as cognitive impairments such as diminished vigilance (Blasini et al., 2017), perceived accuracy (Horváth et al., 2024), and reaction time (Benedetti et al., 2003). Such evidence reveals that negative expectations can objectively diminish various aspects of human performance, thereby engendering self-reinforcing cycles of failure in the domains of motor, perceptual, and cognitive function.

In the case of everyday physical activities and functional autonomy, the nocebo effect may have significant implications with potential consequences for movement, independence, and quality of life (Russell et al., 2022; Colloca and Miller, 2011; Häuser et al., 2012; Horváth et al., 2021). However, research on its impact in these areas remains mixed, particularly regarding objective measures such as joint kinematics, balance and coordination (Lamoth et al., 2004; Daneau et al., 2021; Russell et al., 2022; Horváth et al., 2024). Furthermore, the existing literature is predominantly focused on athletic populations, making it difficult to extrapolate conclusions to the general population engaging in everyday activities. Such findings raise concerns about how nocebo-driven beliefs may constrain physical capabilities for individuals, affecting their quality of life and independence.

Despite growing interest in the nocebo effect within clinical and sports settings, its specific impact on objective motor outcomes in healthy individuals remains underexplored. While Horváth et al. (2021) provided a broad review of nocebo effects across various populations and tasks, their synthesis did not isolate the impact on strictly quantifiable movement parameters, such as three-dimensional kinematics (e.g., joint coordination variability), kinetic outputs (e.g., ground reaction forces), spatiotemporal precision (e.g., gait or sprint timing), or sustained performance in healthy individuals. This scoping review aims to address that gap, and respond to the research questions:

- *What methods are used to induce the nocebo effect in motor performance?*
- *How is the nocebo effect measured in this context?*
- *What are the observed effects on objective motor outcomes?*

## Materials and methods

### Design

This review was drafted using the Preferred Reporting Items for Systematic Reviews and Meta-analysis Protocols Extension for Scoping Reviews guidelines (PRISMA-ScR) (Tricco et al., 2018), and its search strategy was reviewed by an expert librarian and coauthors using the Peer Review of Electronic Search Strategies (PRESS) checklist (McGowan et al., 2016) and modified as required.

### Search strategy

Studies were identified through searches in the databases: PubMed, EBSCO, EMBASE, Cochrane, Web of Science, Scopus, and Google Scholar. Search was conducted from database inception (no lower date limit) until March 6th, 2025. As keywords, terms related to the population “healthy adults,” the intervention “nocebo” and the outcome “motor performance” were used (Table 1) by combining the search strings (*Population AND Intervention AND Outcome*). The search strategy was adapted to each database’s specific requirements, including controlled vocabulary (e.g., MeSH terms in PubMed) and syntax rules (e.g., wildcard characters). These modifications were implemented under the supervision of a librarian to ensure optimal retrieval. The search engine was set to find the keywords in the title, abstract, or keywords provided by the authors through all the different databases. Additional studies were added by other sources (e.g., cross-reference; previous reviews). The full research strategy is provided in Supplementary Table.

**Table 1:** Search strategy.

Search terms	Search strategy
Population	(“Adult*”) AND (“Healthy” AND (“Volunteer*” OR “Participant*” OR “Individual*” OR “Subject*” OR “Control*”))
Nocebo	(“Pain” OR “Noxious Stimul*” OR “Neutral stimul*” OR “Negative expectation” OR “Nocebo respon*”) AND (“Pain Expectation” OR “Anticipat*” OR “Conditioning” OR “Fear”)
Motor performance	“Gait” OR “Walking” OR (“Physic*” OR “Behavior*” OR “Postur*” OR “Movement*” OR “Ambulat*” OR “Locomot*”) AND (“Adaptat*” OR “Compensat*” OR “Adjust*” OR “Activit*”)

### Study selection

The Covidence Systematic Review Software (Veritas Health Innovation), a web-based collaboration platform for managing systematic and literature reviews, was used. During the *title and abstract screening* phase, articles were evaluated by two independent reviewers, with advancement to full-text review requiring mutual agreement on its relevance. Discrepancies were resolved through structured discussion between the original reviewers, followed by arbitration from a third reviewer when consensus could not be reached.

### Eligibility criteria

- Studies were required to be published as full-length articles; abstracts, reviews, thesis dissertations, book chapters, and double publications were not considered. The determination of the inclusion criteria was based on the PICO (population, intervention, control, outcome) standard. The following criteria were applied.
- Studies were restricted to healthy adults aged 18–65 years without mobility impairments or clinical conditions that could confound motor performance (e.g., neurological, musculoskeletal, or psychiatric disorders). This ensured observed effects could be attributed solely to nocebo interventions rather than underlying pathologies. Multi-arm trials involving both healthy and clinical populations were retained *only* if they reported separable data for healthy subgroups (e.g., healthy baseline vs. healthy nocebo), enabling isolated analysis of the target population.
- The study needed to include a nocebo intervention deliberately designed to elicit negative expectations about motor performance or physical sensations. Excluding general negative outcomes not intended to cause a nocebo effect (e.g., reading adverse effects on a drug).
- Articles must have at least one objectively quantifiable movement parameter to isolate the effect of nocebo on movement execution, such as motion characteristics, strength, temporal and spatial precision, endurance, etc. For instance, muscle activation or subjective scales as perceived effort were not included.
- The study design had to incorporate a comparator allowing the isolation of the nocebo’s effect by comparing the nocebo-treated state to another that did not receive the nocebo induction, even if they were exposed to the same treatment without the nocebo element. The nocebo effect was then calculated as the difference between the 2 conditions.

### Data charting

To facilitate analysis, a structured data charting table was developed to systematically record key study characteristics. The following data were collected:

- Study reference: Source ID, authors, publication year, country, and study design.
- Population: Sample size, sex distribution (% male/female), mean age and standard deviation, and population characteristics (e.g., athletes, physically active individuals).
- Intervention: Study aim, motor task performed, control condition or comparator used in the study, and statistical analysis method.
- Nocebo agent: Substance, treatment, or procedure that, when administered to the participant, was intended to lead to negative outcomes or worsen symptoms due to the participant’s negative expectations or beliefs about it.
  - Time of application respect to the nocebo agent administration
  - Type—divided based on the administration format:
    - Oral, substances in pill, capsule or drink format.
    - Topical, external substances applied to skin or body surface (e.g., cream).
    - Injection/Infusion, introduced via needle (e.g., intravenous injections).
    - Electrophysical, electrophysical agent interacting with the body with a device or energy-based stimuli (e.g., thermal probes).
    - Other, unconventional methods not covered by the above categories.
  - Active or Sham, specifies whether the administered agent contained physiologically active components capable of directly influencing participant performance (*active*) or was an inert treatment designed to be neutral (*sham*).
- Induction method—divided into:
  - Verbal instruction, for explicit negative instructions given verbally, written or in audio format (e.g., telling an athlete a pill will reduce strength).
  - Visual cues, for videos, images or demonstrations to imply harm (e.g., showing a video of an athlete struggling after taking a supplement).
  - Conditioning, for studies in which a neutral stimulus is repeatedly paired with a covertly manipulated negative motor experience (e.g., secretly increasing resistance on a leg press machine after administering a sham “fatigue-inducing” spray).
  - Combined methods, for studies merging multiple techniques.
- Target instruction: intended effect of nocebo induction (e.g., provoking pain, anxiety, motor impairment)
- Protocol summary: brief description of the protocol and nocebo administration process.
- Measures/Outcomes: Primary and secondary outcomes, equipment and measurement details (e.g., brand, sampling frequency).
- Main results: Whether the participants were expecting to experience a nocebo effect (“Nocebo effect expected”), whether there is an actual observed effect (“Nocebo effect observed”), the effect size (if reported), and key findings.

Microsoft Excel (v. 2,505 Build 16.0.18827.20102) was used to calculate descriptive statistics (e.g., totals, percentages) and to generate figures to summarize the data.

## Results

### Article selection

**Figure 1:**
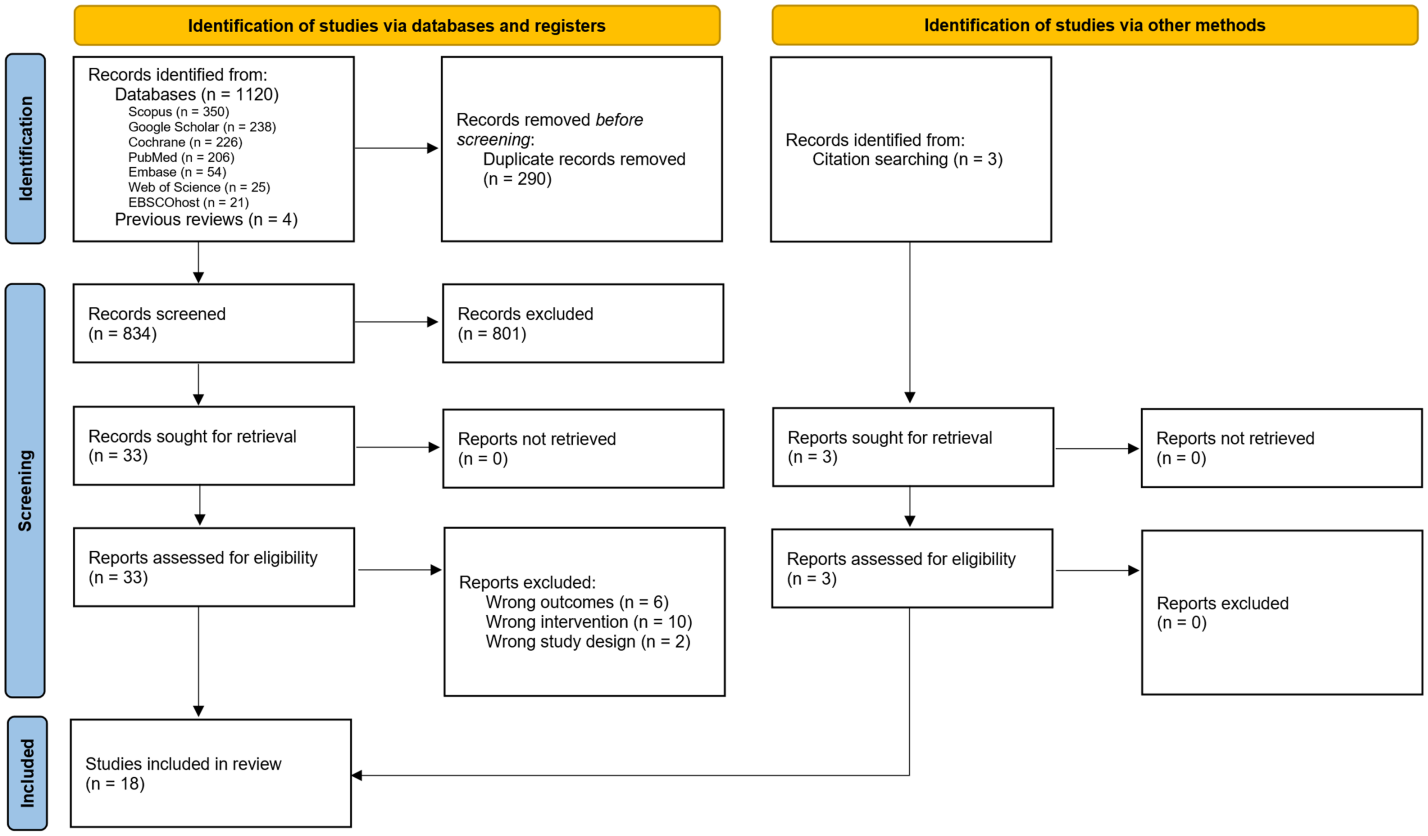
PRISMA flow diagram of the study selection process.

The systematic search, using the predefined search strategy, yielded 1.120 articles, with 834 remaining after duplicate removal. Title and abstract screening excluded 801 records that did not meet inclusion criteria pertaining to population characteristics, nocebo intervention parameters, or objective motricity measurements. The remaining 33 articles underwent full-text assessment, resulting in the exclusion of 18 studies due to wrong interventions (*n* = 10), wrong outcome measures (*n* = 6), or wrong study designs (*n* = 2), with respect to the inclusion criteria. Fifteen articles satisfied all eligibility criteria, with three additional studies (Bottoms et al., 2014; Hurst et al., 2017; Russell et al., 2022) identified through manual reference checking. Consequently, 18 studies were included in the final review. The selection process is documented in the PRISMA flow diagram (Figure 1), with all included studies subjected to standardized data charting procedures as outlined in the methodology section. Although Daneau et al. (2021) studied individuals with and without chronic back pain, their outcomes were analyzed separately, making the healthy subgroup eligible for inclusion.

### Characteristics of included articles

**Figure 2:**
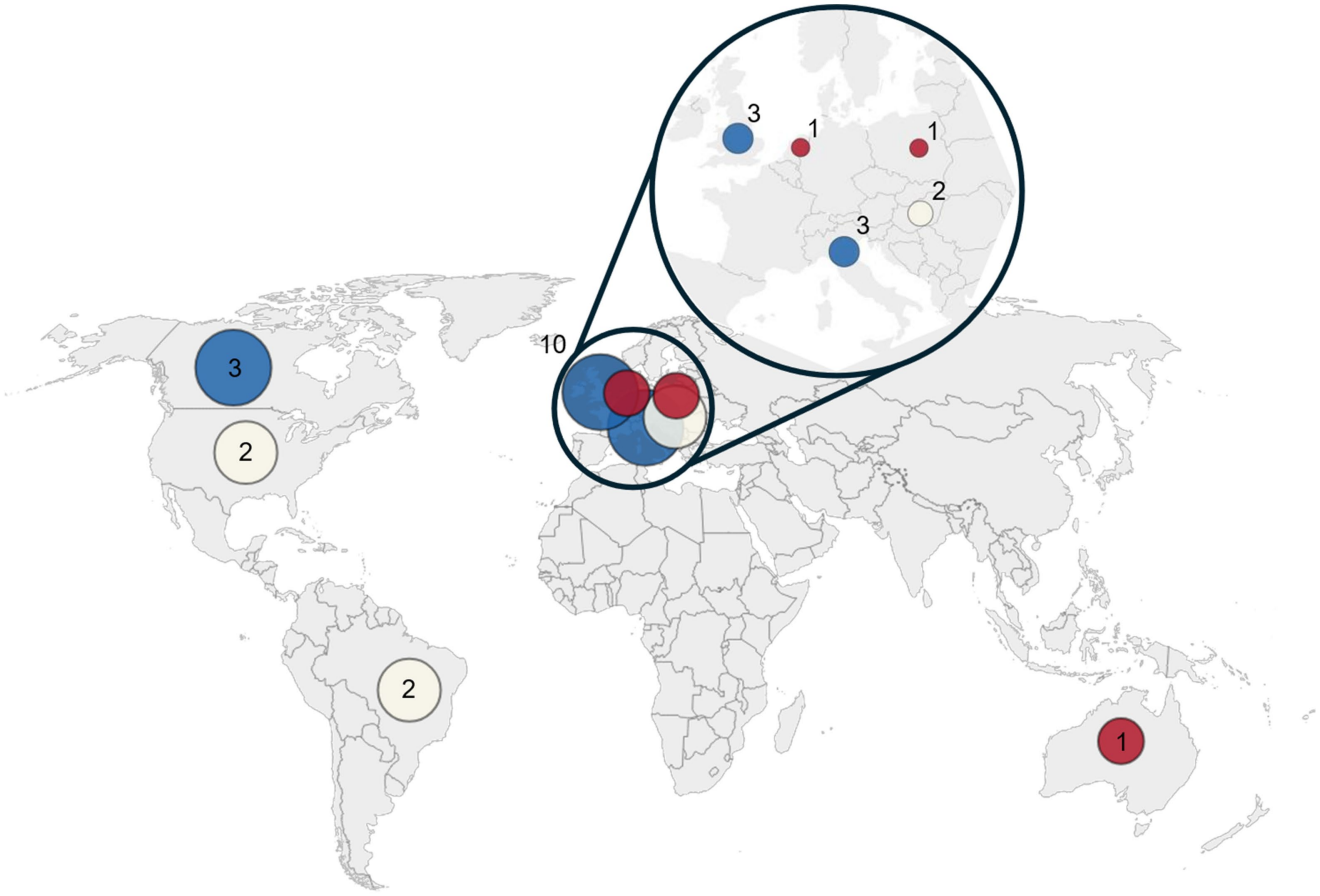
Distribution of articles published. Each circle represents the number of articles published per country (red = 1 article, cream = 2 articles, blue = 3 articles). The inset highlights the European region, where most publications were concentrated. The world map base in figure is adapted from a work by Vardion, vectorized by Simon Eugster (2006), available under a Creative Commons Attribution-Share Alike 3.0 Unported license (CC BY-SA 3.0). The original work can be found at https://commons.wikimedia.org/wiki/File:BlankMap-World_gray.svg. Modifications were made by JB-T to add labels and an inset.

All articles were published in English between 2004 and 2024, with contributions from research teams in Italy (17%), Canada (17%), United Kingdom (17%), Brazil (11%), Hungary (11%), United States (11%), and other countries (Poland, Australia, Netherlands) (Figure 2).

The characteristics of the final articles included are reported in Table 2. These studies included a total of 1.260 healthy participants; 62.8% were male, 32.4% were female, and 4.8% did not report the sex of the participants (Beedie et al., 2007; Daneau et al., 2021). The mean age across studies—the average of the average ages in the studies—was 24.5 ± 6.0 years, excluding Hurst et al. (2017), which reported only the age range (18–44) and Lamoth et al. (2004) which provided both the mean and the range but no standard deviation (mean = 21, range between 18 and 25). Six out of the 18 articles recruited participants with additional criteria beyond general health (Beedie et al., 2007; Pollo et al., 2012; Hurst et al., 2017; McLemore et al., 2020; Campelo et al., 2023; Zagatto et al., 2024), such as being physically active or athletes, while one specifically sought untrained males (≤ 1 day of resistance training per week) (McLemore et al., 2020).

**Table 2:** Characteristics of the included studies in chronological order.

Source ID	Authors	Year	Design	Sample size	% Male	% Female	Age	SD	Characteristic(s) (if any)	Task	Type	Sham/Active	Induction method	Target / Instruction	Outcome(s)	Secondary Outcome(s)	Nocebo effect expected	Nocebo effect observed	Effect size (if reported)
(1)	Lamoth, C. et al.	2004	WS	12	66.7%	33.3%	21.0			Treadmill walking at different velocities.	Injection/Infusion	Sham	Verbal instruction	Induce fear of pain	Trunk coordination, EMG	Variability in movement and muscle activity	Yes	No	NR
(2)	Beedie, C. J., et al.	2007	WS	42	NR	NR	19.6	2.9	Team-sport athletes	3 × 30-m repeat-sprint	Oral	Sham	Verbal instruction	Worsen the performance	Sprint time (speed)	Beliefs on the treatment	No	Yes	NR
(3)	Tétreau, C. et al.	2012	WS	22	54.5%	45.5%	30.1	9.0		Flexion and extension of the trunk from a standing position	Electrophysical	Sham	Combined methods: verbal instruction, conditioning	Induce an anticipation of pain	Pain perception, sEMG, lumbopelvic kinematic variables (hip angle, lumbar angle, lumbar/hip (L/H) angle ratio and lumbar and hip range of motion)	NR	NR	Yes	NR
(4)	Pollo, A., et al.	2012	BS	70	100%	0%	20.5	22.9	Recreationally active students	Leg extension to exhaustion at 65% 1RM	Electrophysical	Sham	Combined methods: verbal instruction, conditioning	Decrease performance	Total work, repetitions	Rate of perceived exertion (RPE, Borg scale)	NR	Yes	NR
(5)	Bottoms L, Buscombe R, Nicholettos A.	2013	WS	12	100.0%	0.0%	25.3	4.4		Pedal at 70 rev. min at increasing workloads.	Oral	Sham	Verbal instruction	Induce fatigue	Peak Minute Power (PMP) and perceived exertion.	Oxygen consumption (V O2), respiratory exchange ratio (RER), carbon dioxide production (V CO2) and minute ventilation (VE), heart rate, perceived exertion of local muscles.	Yes	No	NR
(6)	Hodges, P., Tsao, H., Sims, K.	2015	WS	10	100.0%	0.0%	28.0	6.0		Step down from a step onto a force plate.	Electrophysical	Active	Conditioning	Induce an anticipation of pain	Hip extensor muscle EMG	Peak vertical ground force	NR	No	NR
(7)	Corsi, N., Emadi Andani, M., Tinazzi, M. et al.	2016	WS	41	56.1%	43.9%	22.7	3.1		Abduction of the index finger against a force transducer.	Electrophysical	Sham	Combined methods: verbal instruction, visual cues	Reduce the force and increase the fatigue	Mean value of peak force amplitude (Normalized Forcepeak), percentage of strong pressures (Strongpress), and perception of treatment effectiveness (*Δ* TENS effectiveness)	Subjective measures (TENS efficacy scores, perception of force, sense of effort, expectation).	Yes	Yes	η^2^ = 0.456 (Force peak) η^2^ = 0.295 (Strong press)
(8)	Hurst, P. et al.	2017	BS	527	78%	22%	18–44		Competitive athletes	5 × 20-m sprint	Oral	Sham	Verbal instruction	Decrease the sprint speed	Sprint time (speed)	Likelihood of usage of supplements	NR	Yes	d = 0.32
(9)	Corsi, N., et al.	2019	WS	53	47%	53%	22.3	2.5		Abduction of the index finger against a force transducer.	Electrophysical	Sham	Combined methods: verbal instruction, conditioning	Worsen the force executed	Mean value of peak force amplitude (Normalized Forcepeak), percentage of strong pressures (Strongpress), and perception of treatment effectiveness (Δ TENS effectiveness)	Motor evoked potentials amplitude, cortical silent period, expectation of performance, perceived efficacy of TENS	Yes	Yes	baseline/verb-cond-: d = 0.153 (force peak) d = 1.452 (normalized force peak)
(10)	McLemore, B. H., et al.	2020	BS	14	100%	0%	20.5	0.9	Untrained males (≤ 1 day of resistance training per week)	Eccentric bicep curl at 60 bpm.	Oral	Sham	Verbal instruction	Increase blood flow and inflammation leading to increase muscle soreness and exercise performance	Muscle soreness, range of motion (ROM), ratings of perceived exertion	Total repetitions, belief questionnaire	Yes	Yes	d = 1.83 (ROM) d = 2.51 (repetitions)
(11)	Daneau, C., et al.	2021	WS	19	NR	NR	29.2	8.9		Freestyle lifting of boxes.	Other	Sham	Combined methods: verbal instruction, visual cues	Modulate participants’ expectations about the boxes weight	Kinematics (time to maximal flexion, angular velocity and joint angles), EMG, MVC, CoP, perceived exertion.	Questionnaires on fear-avoidance beliefs (FABQ), pain catastrophizing (PCS), kinesiophobia (TSK), and state–trait anxiety (STAI-Y) were collected.	Yes	Yes	NR
(12)	Russell, K., et al.	2022	BS	42	52%	48%	21.4	2.2		Stand stable in bipedal and unipedal stances.	Oral	Sham	Verbal instruction	Reduce alertness and cause sensation of fatigue, lethargy and tiredness	Posturography (Anterioposterior and mediolateral COP range) and perceived postural stability	Subjective performance expectation perceived change in performance and advverse symptoms	Yes	Yes	Bipedal: d = 1.11 (anterioposterior) d = 0.47 (mediolateral) Unipedal: d = 1.11 (anterioposterior) d = 1.61 (mediolaterail)
(13)	Horváth, Á., Szabo, A., Gál, V. et al.	2023	BS	78	26.9%	73.1%	20.7	3.3		Stand stable in various sensory modalities (standard, vision, proprioception and vestibular).	Topical	Sham	Verbal instruction	Decrease balancing ability.	Total COP Path Length Index	Expected and perceived performance	Yes	No	standard: d = 0.501 proprioceptive: d = 0.330 visual: d = 0.590 vestibular: d = 0.155
(14)	Campelo D, Koch AJ, Machado M.	2023	WS	15	100.0%	0.0%	41.0	4.0	Trained men, experienced in resistance training	Bench press at 80% of their one-repetition maximum test (1RM) until failure	Oral	Sham	Combined methods: verbal instruction, visual cues	Cause muscle fatigue and hamper performance	Repetitions completed until failure and rating of perceived exertion	NR	Yes	No	d = 0
(15)	Zaworski, K., Kadlubowska, M., Baj-Korpak, J.	2023	BS	88	38.6%	61.4%	22.6	5.2		Hold the grip of a dynamometer as tight as possible for 3 s	Topical	Sham	Verbal instruction	Decrease hand muscle strength	sEMG and hand grip strength	NR	No	No	NR
(16)	Horváth, Á. Aranyosy, B., et al.	2024	BS	78	17%	83%	21.3	3.9		Joint position reproduction test (active/passive elbow joint movement)	Electrophysical	Sham	Verbal instruction	Worsen the proprioceptive accuracy	Actual accuracy (absolute error in joint angle reproduction)	Perceived performance change (VAS), state anxiety (STAI-S), optimism (LOT-R)	Yes	No	NR
(17)	Hanson, N. J., Maceri, R. M. & Koutakis, P.	2024	WS	23	39%	61%	25.0	6.2		3-min aerobic test (3mAT) on cycle ergometer - maintaining highest power output for 3 min	Electrophysical	Active	Combined methods: verbal instruction, visual cues	Hinder performance	Maximal Minute Power (MMP), VO2 max, Peak HR	Rating of Perceived Exertion (RPE), post-study belief questionnaire	No	No	η^2^ = 0.002
(18)	Zagatto, A. M., Lopes, V. H. F., Dutra, Y. M. et al.	2024	WS	14	100.0%	0.0%	26.0	7.0	Physically active (i.e., engage in physical activity at least 3 times per week)	Constant-load cycling time-to-task failure at 115% of peak power output.	Oral	Sham	Combined methods: verbal instruction, visual cues	Impair physical performance	Time to task failure, VO2max	blood lactate, blood acid–base balance, vastus laterallis muscle activity (iMVC)	No	No	d = 0.27

BS, between-subject design. WS, within-subject design. NR, not reported.

Eight out of 18 studies (44.4%) focused on sports performance (Beedie et al., 2007; Pollo et al., 2012; Bottoms et al., 2014; Hurst et al., 2017; McLemore et al., 2020; Campelo et al., 2023; Hanson et al., 2024; Zagatto et al., 2024), with five of these studies specifically requesting trained and active individuals (Beedie et al., 2007; Pollo et al., 2012; Hurst et al., 2017; Campelo et al., 2023; Zagatto et al., 2024). The remaining articles (55.5%) had a more varied focus, including applications such as walking (Lamoth et al., 2004), joint movement (Tétreau et al., 2012; Corsi et al., 2016; Corsi et al., 2019; Horváth et al., 2024), standing (Russell et al., 2022; Horváth et al., 2023), stepping down (Hodges et al., 2015), lifting boxes (Daneau et al., 2021), and strength exercises (Zaworski et al., 2023), that could be incorporated into activities of daily living.

### Nocebo intervention characteristics

A summary of the number of induction methods to deliver the information and their time of induction in respect to the nocebo agent is provided in Table 3. The most common nocebo induction method was verbal instruction, used in 17 studies (94.4%). Nine studies used verbal instruction exclusively (Lamoth et al., 2004; Beedie et al., 2007; Bottoms et al., 2014; Hurst et al., 2017; McLemore et al., 2020; Russell et al., 2022; Horváth et al., 2023; Zaworski et al., 2023; Horváth et al., 2024), specifically verbal cues, with information delivered pre-nocebo agent application (Lamoth et al., 2004; Horváth et al., 2023; Horváth et al., 2024) or post-nocebo agent application (Beedie et al., 2007; Bottoms et al., 2014; Hurst et al., 2017; McLemore et al., 2020; Russell et al., 2022; Zaworski et al., 2023).

**Table 3:** Summary of usage of nocebo induction methods, timing relative to the nocebo agent administration and success rate.

Induction method	Total studies	Nocebo effect observed^a^	Time of the induction		Success rate^b^
			Before	After	
Combined methods: verbal instruction, visual cues	5	2	2	3	40.0%
Verbal instruction	9	4	3	6	44.4%
Combined methods: verbal instruction, conditioning	3	3	1	2	100.0%
Conditioning	1	0	1	0	0.0%

^a^Significant nocebo effect defined as *p* < 0.05 in intervention vs control condition comparisons.

^b^Success rate calculated as (Nocebo effect observed / Total studies) × 100.

The remaining eight studies combined the following:

- Verbal plus visual cues (*n* = 4) (Corsi et al., 2016; Daneau et al., 2021; Campelo et al., 2023; Zagatto et al., 2024), where information timing varied [pre-agent application only (Corsi et al., 2016; Daneau et al., 2021; Zagatto et al., 2024) or post-agent application (Campelo et al., 2023)];
- Written plus visual cues (*n* = 1) (Hanson et al., 2024), pre-agent application; and
- Verbal cues plus conditioning (*n* = 3) (Tétreau et al., 2012; Pollo et al., 2012; Corsi et al., 2019) with information delivered either pre-agent (Tétreau et al., 2012) or post-agent (Pollo et al., 2012; Corsi et al., 2019).

Conditioning was implemented as a standalone intervention in only one study (Hodges et al., 2015), with information given before the nocebo agent.

The nocebo agent varied across studies, with *oral ingestion* (39%) and *electrophysical stimulations* (39%) being the most frequently administered type. Oral substances included cornstarch, sodium bicarbonate and sugar-free drinks (Beedie et al., 2007; Bottoms et al., 2014; Hurst et al., 2017; McLemore et al., 2020; Russell et al., 2022; Campelo et al., 2023; Zagatto et al., 2024), while stimulations comprised device-based interventions such as electrical stimuli like transcranial direct current stimulation and transcutaneous electrical nerve stimulation as well as noxious electrical and thermal stimuli (Tétreau et al., 2012; Pollo et al., 2012; Hodges et al., 2015; Corsi et al., 2016; Corsi et al., 2019; Horváth et al., 2024). *Topical applications* represented a smaller proportion of studies (11%), using the application of inert cream and paper tape. Less common administration routes included *injection* (6%) with an isotonic saline injection (Lamoth et al., 2004) and *others* (6%) with the use of altered visual signs (Daneau et al., 2021), each of these methods being represented in a single study. Among the 18 studies reviewed, 16 used sham treatments while two employed active treatments (Hodges et al., 2015; Hanson et al., 2024).

### Motor task and measurements

The studies assessed a variety of motor tasks, each with distinct kinematic and performance outcomes. *Flexion and extension* of the trunk and leg, and *eccentric bicep curl* were used to measure joint angles, angle ratios, range of motion, work output, and repetition counts (Tétreau et al., 2012; Pollo et al., 2012; McLemore et al., 2020). *Index finger abduction* against a force transducer provided data on peak force amplitude and repetitions (Corsi et al., 2016; Corsi et al., 2019)*. Box lifting tasks* was used to evaluate kinematic efficiency through metrics such as time to maximal flexion, angular velocity, joint angles, and center of pressure displacement (Daneau et al., 2021). Strength endurance was assessed via *bench press* tests, recording repetitions until failure (Campelo et al., 2023). *Cycling and arm pedalling* measured time to task failure and maximal power output (Bottoms et al., 2014; Hanson et al., 2024; Zagatto et al., 2024).

Tasks included *stepping-down* exercises, which tracked peak vertical ground reaction force (Hodges et al., 2015), and *treadmill walking*, which analysed trunk coordination and movement variability (Lamoth et al., 2004). *Sprint performance* which was quantified by sprint time (Beedie et al., 2007; Hurst et al., 2017), and *postural stability* which was assessed using posturography to measure center of pressure (COP) displacements (Russell et al., 2022; Horváth et al., 2023). A *joint position reproduction test*, was also used to assess the accuracy in angle replication (Horváth et al., 2024) and *grip endurance* was evaluated via dynamometer-based hand strength measurements (Zaworski et al., 2023).

Most of the tasks belong to the group of gross motor tasks (*n* = 15), particularly with *lower-limb* (Lamoth et al., 2004; Beedie et al., 2007; Bottoms et al., 2014; Hodges et al., 2015; Hurst et al., 2017; Hanson et al., 2024; Zagatto et al., 2024; Benedetti et al., 2003) and *whole-*body (Tétreau et al., 2012; Daneau et al., 2021; Russell et al., 2022; Horváth et al., 2023) movements such as cycling, sprinting, and postural control and upper-limb movements, such as bench press (Campelo et al., 2023), eccentric bicep curl (McLemore et al., 2020), and the maintenance of a dynamometer grip (Zaworski et al., 2023). Fine motor tasks (*n* = 3) included only upper-limb movements such as finger abduction (Corsi et al., 2016; Corsi et al., 2019) and employed a joint position sense task (Horváth et al., 2024).

**Figure 3:**
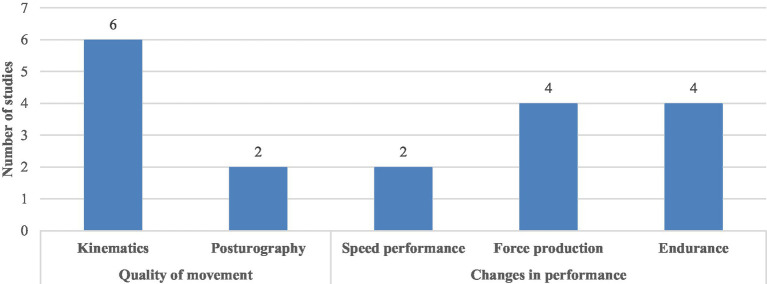
Distribution of the metric in the outcomes.

The motor tasks evaluated in these studies encompassed key biomechanical and performance dimensions (Figure 3), which can be categorized into five core concepts:

- Kinematics: assessments that included joint angles, range of motion, angular velocity, trunk coordination, and movement variability (Lamoth et al., 2004; Tétreau et al., 2012; Pollo et al., 2012; McLemore et al., 2020; Daneau et al., 2021; Horváth et al., 2024)
- Posturography: postural stability quantified through COP displacement measurements (Russell et al., 2022; Horváth et al., 2023).
- Speed Performance: such as sprint time (Beedie et al., 2007; Hurst et al., 2017).
- Force production: including peak force amplitude, strength endurance (Hodges et al., 2015; Corsi et al., 2016; Corsi et al., 2019; Zaworski et al., 2023).
- Endurance: (repetitions until failure), maximal power output, and grip endurance (Pollo et al., 2012; Campelo et al., 2023; Hanson et al., 2024; Zagatto et al., 2024).

### Measured effects

**Figure 4:**
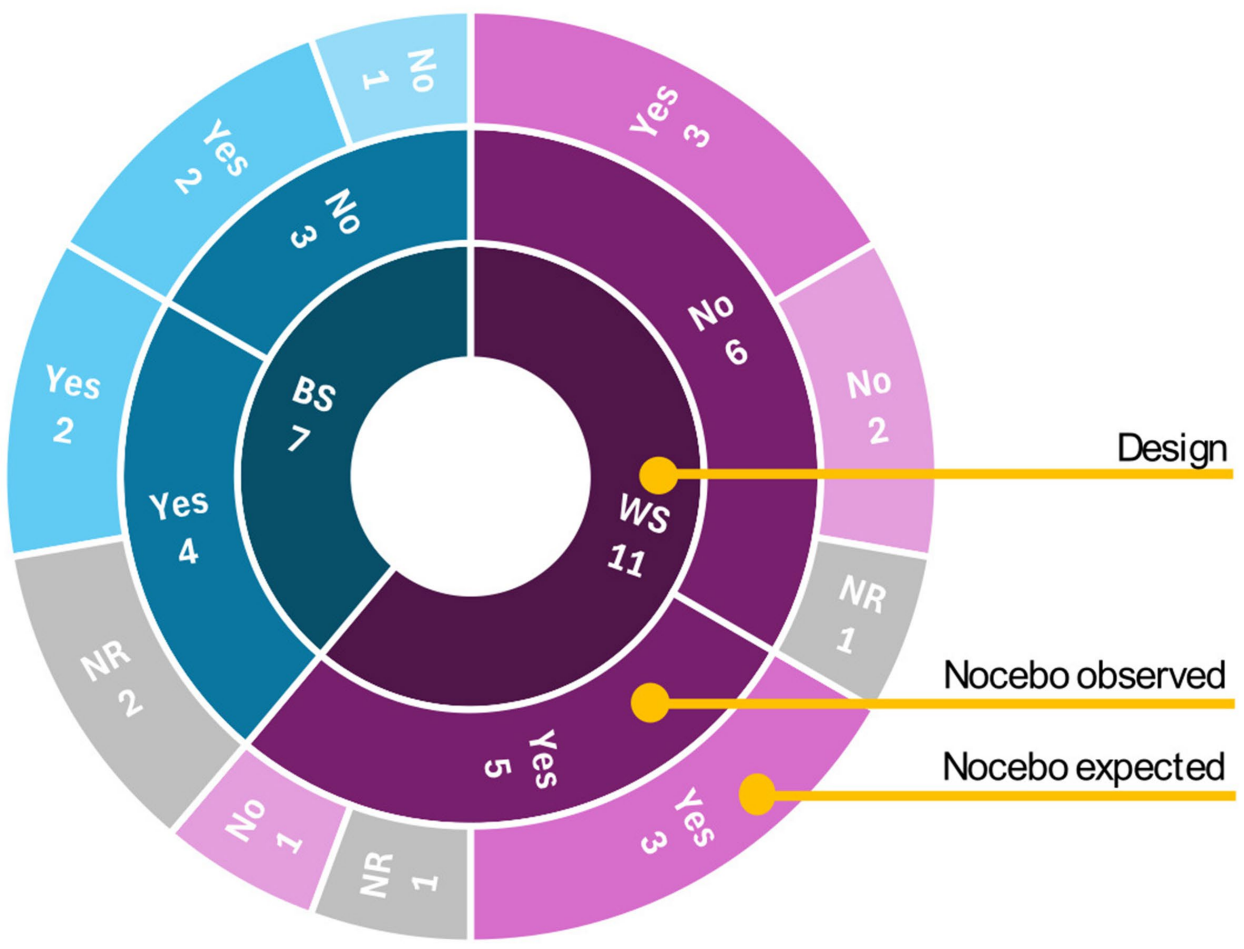
Sunburst graph of nocebo effect studies: distribution by study design (inner), confirmed effects (middle), and participants’ belief of experiencing nocebo effects (outer). WS, within-subject; BS, between-subject; NR, not reported.

Figure 4 displays a three-level sunburst graph, each level representing a distinct layer of the data. In general, nine out of the 18 studies confirmed having a nocebo effect in the measured outcomes, while the remaining nine did not manifested obtaining a nocebo effect.

The articles included eleven studies using within-subject designs and seven using between-subject designs. Among within-subject studies, five reported statistically confirmed nocebo effects including reduced force exertion (Corsi et al., 2016; Corsi et al., 2019), decreased range and time of motion (Tétreau et al., 2012; Daneau et al., 2021), and impaired sprint performance (Beedie et al., 2007). In these five confirmed-effect studies, three reported participants’ expectations of worsened outcomes, and one did not report participants’ opinions. Among the six within-subject studies without confirmed effects, three still reported the expected worsening outcome.

Among the seven between-subject studies, four reported statistically confirmed nocebo effects including decreased range of motion and repetitions until failure (Pollo et al., 2012; McLemore et al., 2020), decrease in total work (Pollo et al., 2012), increased sway (Russell et al., 2022) and sprint time (Hurst et al., 2017), while three showed no significant effects when measuring proprioceptive accuracy (Horváth et al., 2024), hand strength (Zaworski et al., 2023) and balance (Horváth et al., 2023). Of the five confirmed-effect studies, two documented participants’ expectations of worsened outcomes, one reported no such expectations, and two did not report participants’ opinions. Both studies without confirmed effects reported expected worsened outcomes despite the absence of statistical significance.

## Discussion

The purpose of this scoping review was to examine the methodologies employed to induce nocebo effects in motor performance and to evaluate their impacts on quantifiable movement parameters in healthy individuals. A total of 18 articles reporting findings on the nocebo effect across several motor tasks were identified and examined for this review.

The Nocebo intervention characteristics, the Motor task and measurements, and the Measured effects are presented below to give responses to the questions addressed in this scoping review.

### Nocebo intervention characteristics

The first objective of this scoping review was to examine the methodologies used to induce negative expectations in healthy individuals. For this review, nocebo interventions were defined as intentional manipulation of information or environmental factors intended to elicit negative expectations or associations with the aim of affecting the motricity. The results related to this first research question are discussed in the following subsections, distinguishing between two key components: the *modalities of information transmission*, referring to how negative expectations are conveyed (e.g., verbal warning, visual cues); and the *nocebo agent*, defined as the tool used to create or reinforce the expectations (e.g., placebo pills, sham treatments).

### Modalities of information transmission

Three modalities of information transmission were identified: verbal instructions, visual cues, and conditioning. These can be divided into two pathways guiding nocebo effects: instructional learning (explicit, expectation-based) and associative learning (implicit, experience-based). The most common learning pathway was instructional learning (*n* = 17) (Lamoth et al., 2004; Beedie et al., 2007; Pollo et al., 2012; Tétreau et al., 2012; Bottoms et al., 2014; Corsi et al., 2016; Hurst et al., 2017; Corsi et al., 2019; McLemore et al., 2020; Daneau et al., 2021; Russell et al., 2022; Horváth et al., 2023; Campelo et al., 2023; Zaworski et al., 2023; Horváth et al., 2024; Hanson et al., 2024; Zagatto et al., 2024). Among these 17 studies, three (Tétreau et al., 2012; Pollo et al., 2012; Corsi et al., 2019) combined instructional learning with associative learning. Only one article (Hodges et al., 2015) used associative learning by itself.

*Instructional learning* involves conscious expectations formed through direct communication or observation (Colloca and Barsky, 2020; McGowan et al., 2016; Stewart-Williams and Podd, 2004; Manaï et al., 2019), and was primarily implemented through verbal instruction - either as a standalone intervention (9 out of 17) (Lamoth et al., 2004; Beedie et al., 2007; Bottoms et al., 2014; Hurst et al., 2017; McLemore et al., 2020; Russell et al., 2022; Horváth et al., 2023; Zaworski et al., 2023; Horváth et al., 2024) or in combination with other methods (8 out of 17), such as visual cues or conditioning (Tétreau et al., 2012; Pollo et al., 2012; Corsi et al., 2016; Corsi et al., 2019; Daneau et al., 2021; Campelo et al., 2023; Hanson et al., 2024; Zagatto et al., 2024).

In contrast, *associative learning* operates through implicit experience-driven associations where a neutral stimulus is paired with a negative outcome, creating subconscious predictions and nocebo responses without verbal warnings (Colloca and Barsky, 2020; Klauß et al., 2024; McGowan et al., 2016; Stewart-Williams and Podd, 2004; Manaï et al., 2019). A clear example is demonstrated by Hodges et al. (2015) where participants stepped down from a platform. Foot contact with the ground triggered a painful electrical stimulus. After repeated pairings of the step-down task (neutral stimulus) with pain (negative outcome), participants exhibited anticipatory postural adaptations even in trials where pain was *expected but not delivered*. This mirrors classic associative learning paradigms, as the motor system generalized the conditioned response (augmented postural “gain”) to contexts where pain was anticipated but absent.

Instructional learning, specifically verbal instruction, were more likely to demonstrate nocebo effects when paired with complementary techniques. Among combined-method studies, 5 out of 8 (62.5%) reported statistically significant (vs. control conditions) nocebo effects (Tétreau et al., 2012; Pollo et al., 2012; Corsi et al., 2016; Corsi et al., 2019; Daneau et al., 2021), compared to 4 out of 9 studies (44.4%) using verbal instruction alone (Beedie et al., 2007; Hurst et al., 2017; McLemore et al., 2020; Russell et al., 2022). However, methodological differences across studies, such as the intended target (e.g., pain vs. performance) or task design (e.g., static vs. dynamic movements), can also influence nocebo effects. Hence, while combined learning pathways appear to produce more robust effects, variations in experimental paradigms could also contribute to the observed differences in these success rates. Further research directly comparing combined methods with standalone approaches is needed to clarify their relative effectiveness.

Importantly, verbal instructions can also override the effects of other modalities of transmission of information when conflicting cues are present. For instance, Corsi et al. (2019) employed a combined approach of conditioning and verbal instruction to determine which method would exert a stronger influence. In their study, participants were verbally informed that a sham stimulation would either *increase* (positive verbal instruction) or *decrease* (negative verbal instruction) their force output. At the same time during the experiment, participants also received visual feedback on their force production, with or without surreptitious manipulation of the displayed results (conditioning vs. no conditioning). The findings obtained by Corsi et al. (2019) suggested that the impact of the negative verbal instruction appears to be independent of the conditioning manipulation. However, while Corsi et al. (2019) used live verbal instructions, it remains unclear whether written or multimedia-based warnings would yield similar results.

Interestingly, even verbal instructions unrelated to the measured variable can influence outcomes, suggesting that individuals probably tend to generalize negative expectations based on subjective interpretation rather than treating them as explicit task-related cues. For instance, in the study by Russell et al. (2022), the verbal cue described the nocebo agent as one that would *‘dampen the activity of the central nervous system, reduce* alertness*, and cause sensations of tiredness, fatigue and lethargy’*. In addition to the expected effects reported by Russell et al. (2022) (weakness, drowsiness and fatigue), participants in the nocebo group also exhibited impaired postural control (increased sway), despite this outcome not being directly targeted by the verbal suggestions. A similar pattern emerges in protocols employing nonspecific performance-impairment suggestions (Beedie et al., 2007; Pollo et al., 2012; McLemore et al., 2020; Daneau et al., 2021; Campelo et al., 2023; Hanson et al., 2024; Zagatto et al., 2024) where the experimenter use the expression ‘hinder/worsen the performance’, though with less consistent results: for these cases, nocebo effect was observed only in 57.1% of the studies. While these generalized negative suggestions appear to produce nocebo effects in approximately half of cases, the variability in outcomes suggests that the efficacy of such broad instructions may depend critically on individual differences in expectation formation or contextual factors.

An unresolved question is whether the temporal delivery of the nocebo suggestion—specifically their timing relative to the nocebo agent—modulates their effects. In the reviewed studies, suggestions were mostly administered immediately *after* the application of the nocebo agent (61.1% of the cases), which raises the possibility that alternative timings (e.g., pre-application or even pre-experiment in control groups) might yield different results. For instance, delivering suggestions *before* the administration of the nocebo agent could engage anticipatory mechanisms, potentially amplifying effects compared to post-application suggestions, which may rely on more retrospective symptom interpretation (Hodges et al., 2015; Blasini et al., 2017). Similarly, pre-experiment suggestions (e.g., during study consent) might establish baseline expectations that persist throughout the protocol, parallel to clinical settings where side effect warnings precede treatment (Colloca and Miller, 2011; Grosso et al., 2024).

### Nocebo agents

The reviewed studies demonstrated considerable variation in how nocebo effects were induced. Oral ingestion and electrophysical stimulation were the most common methods (e39% each) (Beedie et al., 2007; Tétreau et al., 2012; Pollo et al., 2012; Bottoms et al., 2014; Hodges et al., 2015; Corsi et al., 2016; Hurst et al., 2017; Corsi et al., 2019; McLemore et al., 2020; Russell et al., 2022; Campelo et al., 2023; Horváth et al., 2024; Zagatto et al., 2024). These observations could be explained by either: (1) a preference for accessible agents, such as pills or drinks, that will likely trigger participants’ expectations based on prior experiences with conventional medications (e.g.*, ‘pills have side effects’*), or (2) sensory-based stimulations like electrical or vibratory inputs that can produce immediate physical sensations, potentially enhancing the idea of a nocebo-related effect (e.g., ‘electrical stimuli are aversive’).

Less common administration routes were topical applications (11%), injection (6%) and the use of altered visual signs (6%). While these methods indicate an effort to simulate a wider range of interventions, their limited use may reflect practical or ethical constraints such as greater risks associated with injections, or weaker pre-existing associations for participants for visual interventions and topical applications compared to oral ingestions and stimulations.

There was a strong predominance of sham treatments (inactive/fake interventions like placebo pills or inert creams; *n* = 16) over active treatments (interventions that produce real physiological effects, such as thermal pain stimuli; *n* = 2) (Hodges et al., 2015; Hanson et al., 2024). While sham interventions are methodologically useful for isolating the nocebo effect, the inclusion of active treatments is also important, particularly to understand how negative expectations might worsen actual treatment outcomes. It is important to consider not only the induction of new symptoms but also the worsening of existing conditions due to nocebo mechanisms. Furthermore, many nocebo effects, such as heightened pain perception or amplified side effects, have direct clinical implications, as they can worsen patients’ subjective and objective outcomes (Nishi et al., 2021; Blasini et al., 2017). Future research should explore how nocebo interventions may modulate patients’ reactions to treatment and the enhancement of already negative experiences.

### Study design

For this scoping review, most studies had a within-subject study design (*n* = 11), which requires the participants to be exposed to the nocebo and the control intervention, usually in a counterbalanced order. Although this design reduces inter-individual variability, it carries the risk of carryover effects, practice and fatigue which may prevent from properly isolating the nocebo effect in the motor task. For instance, repeated performance of a motor task may lead to improved performance over time, even if the participant also experiences negative expectations, thereby confounding interpretation. In the studies (Bottoms et al., 2014; Zagatto et al., 2024), a within-subject design was used with placebo, nocebo and control measurements taken separately over a period of 1 week or less (48 to 72 h), assuming this delay would be enough to prevent carry-over effects. However, pain research demonstrated nocebo effects persist beyond 1 week (Kirsch, 2004; Kunkel et al., 2025), casting doubt on this assumption. As Léonard et al. (2012) emphasize, within-subject designs can artificially inflate or suppress outcomes due to conditioned expectations from prior interventions.

Based on the results from the scoping review, the between-subject design demonstrated a higher chance of success (5 out of 7 studies, 71.5%) than the within-subject design (5 out of 11 studies, 45.4%). Between-subject studies also involved larger sample sizes (mean sample size = 142.4 ± 215.2) than within-subject designs (23.9 ± 14.6), offering greater statistical power and reducing the likelihood of type II errors. Consistent to the review from Horváth et al. (2021) and Léonard et al. (2012) the between-subject design tends to have higher effect sizes and to be more sensitive to catch nocebo effects although it needs a bigger sample size.

### Motor task and measurements

The second objective of this scoping review was to examine how nocebo effects have been assessed in the existing literature. Our findings reveal considerable variation in both the types of tasks used and the outcomes assessed. Lower-limb tasks were the most frequently studied (*n* = 6) (Lamoth et al., 2004; Beedie et al., 2007; Pollo et al., 2012; Hurst et al., 2017; Hanson et al., 2024; Zagatto et al., 2024), including cycling, walking and sprinting, followed by upper-limb tasks (*n* = 8) (Tétreau et al., 2012; Bottoms et al., 2014; Corsi et al., 2016; Corsi et al., 2019; McLemore et al., 2020; Campelo et al., 2023; Zaworski et al., 2023; Horváth et al., 2024), such as bench press, bicep curls, hand gripping, joint position reproduction, and finger abduction. Finally, whole-body movements were the least used (*n* = 4) (Hodges et al., 2015; Daneau et al., 2021; Russell et al., 2022; Horváth et al., 2023), with tasks like stepping down, postural stability and box lifting.

A preference toward gross motor tasks was identified (*n* = 15), particularly with lower-limb and whole-body movements (Lamoth et al., 2004; Beedie et al., 2007; Tétreau et al., 2012; Pollo et al., 2012; Bottoms et al., 2014; Hodges et al., 2015; Hurst et al., 2017; McLemore et al., 2020; Daneau et al., 2021; Russell et al., 2022; Campelo et al., 2023; Zaworski et al., 2023; Hanson et al., 2024; Zagatto et al., 2024). Among the included articles in this review, only three incorporated fine motor assessments: Corsi et al. (2016, 2019) used an index finger abduction task against a force transducer to quantify precision strength modulation in 2 studies, and Horváth et al. (2024) employed a joint position sense task in one study. This imbalance likely reflects methodological convenience, as gross motor skills are probably easier to quantify and align with traditional sports and rehabilitation research which has been assessed before in previous reviews (Hurst et al., 2020; Chhabra and Szabo, 2024). Fine motor skills, on the other hand, require more sensitive measurement tools and are rarely examined, despite their impact and relevance to clinical populations. Notably, most of the articles found in this review were oriented towards sports performance (44.4%) (Beedie et al., 2007; Pollo et al., 2012; Bottoms et al., 2014; Hurst et al., 2017; McLemore et al., 2020; Campelo et al., 2023; Hanson et al., 2024; Zagatto et al., 2024), and included sports-related tasks; in a further five cases (Beedie et al., 2007; Pollo et al., 2012; Hurst et al., 2017; Campelo et al., 2023; Zagatto et al., 2024), the participants were required to be physically active or to have undergone training (Beedie et al., 2007; Pollo et al., 2012; Hurst et al., 2017; Campelo et al., 2023; Zagatto et al., 2024),. The remaining studies (55.5%) examined everyday motor activities (Lamoth et al., 2004; Tétreau et al., 2012; Hodges et al., 2015; Corsi et al., 2016; Corsi et al., 2019; Daneau et al., 2021; Russell et al., 2022; Horváth et al., 2023; Zaworski et al., 2023; Horváth et al., 2024), highlighting a secondary but substantial research focus beyond athletic contexts.

Regarding the motor outcomes measured in the articles included in this review (Figure 3), most of them (10 out of 18) focused on outcomes like the *changes in performance* (i.e., quantifiable decreases in capacity, such as time-to-task failure or maximal force production) over *qualitative changes* (8 out of 18) in movement (i.e., quantifiable deterioration in the movement execution, such as changes in range of motion or center of pressure dynamics). *Qualitative changes* included measurements of accuracy (Horváth et al., 2024), symmetry (Lamoth et al., 2004), stability (Russell et al., 2022; Horváth et al., 2023), time-to-flexion and range of motion (Tétreau et al., 2012; Bottoms et al., 2014; McLemore et al., 2020; Daneau et al., 2021), while the *changes in performance* included strength (Zaworski et al., 2023), power (Hanson et al., 2024), force (Hodges et al., 2015; Corsi et al., 2016; Corsi et al., 2019), endurance (Campelo et al., 2023; Zagatto et al., 2024), work (Pollo et al., 2012) and speed (Beedie et al., 2007; Hurst et al., 2017). Degradations in movement quality, particularly in precision-dependent tasks, may precede, mediate, or amplify declines in performance. For instance, subtle disruptions in trunk coordination or proprioceptive accuracy could initiate a cascade of compensatory adaptations that ultimately manifest as measurable reductions in power output or endurance.

### Measured effects

The third objective of this scoping review was to evaluate the observed effects of nocebo manipulations on motor outcomes. In most of the included studies (10 out of 18), participants explicitly reported believing that their outcomes were influenced by the nocebo effect when questioned post-intervention (*Nocebo expected*, Figure 4). Among these, four studies explicitly reported participants *not* expecting a nocebo effect (Beedie et al., 2007; Zaworski et al., 2023; Hanson et al., 2024; Zagatto et al., 2024), while expectation data was unavailable for the remaining four (Tétreau et al., 2012; Pollo et al., 2012; Hodges et al., 2015; Hurst et al., 2017). Regardless of expectations, observable nocebo effects (statistically significant differences compared to control conditions) were reported in only half of these studies (5 out of 10) for studies assessing endurance, force, posture and kinematics (Corsi et al., 2016; Corsi et al., 2019; McLemore et al., 2020; Daneau et al., 2021; Russell et al., 2022). In most cases when participants did *not* report expecting a negative outcome (3 out of 4), no nocebo effect was observed (Beedie et al., 2007; Zaworski et al., 2023; Hanson et al., 2024; Zagatto et al., 2024). Taken together, these observations suggest that while expectations may be necessary for nocebo effects to occur, they are not always sufficient.

One study demonstrated a particularly interesting dissociation between the participant’s belief and the measured nocebo: despite participants reporting no conscious expectation of performance impairment, the motor assessment revealed significant declines (Beedie et al., 2007). In the study of Beedie et al. (2007) participants had to perform a 30-meter sprint before and after receiving what they believed was a substance that enhances endurance performance while having a negative impact on repeat-sprint performance. At post-intervention, participants in the nocebo group were asked whether they believed the ingested substance had affected their motor task performance. While 67% were unsure about its influence, results demonstrated a decrease on the whole group sprint speed. This finding suggests the involvement of implicit, non-conscious mechanisms in nocebo interventions in motor changes.

In the sham nocebo interventions, nocebo effect was observed in 56.3% of the cases (9/16), with electrophysical and oral methods equally effective (57.1%, 4/7 each), while topical/injection approaches failed (0/3). A nocebo effect (significant change vs. control) occurred in 44.4% of verbal-only studies (4 out of 9), 40% of visual-verbal studies (2 out of 5), and 100% of studies combining conditioning with verbal instruction (3 out of 3), while the only intervention in the “other” category intervention (use of altered visual signs) (Daneau et al., 2021) also demonstrated a significant nocebo effect. These findings suggest that nocebo effects on motor performance are most reliably produced through sham nocebo interventions particularly electrophysical or oral modalities and verbal instructions in combination with a conditioning-based induction.

The failure of nocebo induction through *injection/infusion*—exemplified by Lamoth et al. (2004) null findings (verbal instruction) VS., Tétreau et al. (2012) significant effects (conditioning + noxious heat)—raises the question of whether this is due to the nocebo agent or the type of transmission of information given to the participant. Despite the similarities in their objectives, the two studies diverged in their methodologies. Tétreau’s, for example, included sensory input that reinforced verbal instructions, creating a stronger contextualized expectation, a factor missing in Lamoth’s design, which may explain its lack of efficacy. However, with only two injection studies available, differing protocols and outcome measured prevent definitive conclusions. Future research should dissect these interactions by systematically varying administration routes (topical/injection/oral) and instructional frameworks.

### Limitations

The present scoping review is subject to several methodological and conceptual constraints. Regarding the review process itself, the requirement for quantifiable movement parameters excluded many studies examining nocebo effects through self-report or neurophysiological markers alone, potentially overlooking important psychobiological interactions. Moreover, the heterogeneity in experimental designs, nocebo induction methods, and outcome measures across included studies limited discussions to qualitative patterns rather than quantitative effects.

The reviewed studies predominantly sampled young adults, with a mean age of 24.5 years, limiting the generalizability of findings to older populations or other demographic groups. Additionally, articles from the included research focused essentially on athletes or physically active individuals, raising questions about whether the observed effects can extend to sedentary or untrained populations. Furthermore, given the prevalence of within-subject designs, several studies featured small sample sizes, potentially limiting statistical power to detect subtle nocebo-induced motor impairments. This design choice also raises concerns about carryover effects, practice-related improvements, or fatigue potentially confounding the interpretation of nocebo-specific outcomes.

Finally, the small total number of included studies (*n* = 18) and the even smaller sample sizes within subcategories limit the robustness and generalizability of our conclusions. For instance, definitive claims about the efficacy of specific induction methods (e.g., the 100% success rate of combined verbal-conditioning from only 3 studies) or outcomes (e.g., effects on fine motor control from only 3 studies) are constrained by this scarcity of evidence. The heterogeneity across the included studies further prevented quantitative synthesis, limiting the discussion to qualitative patterns. Therefore, the results presented here should be interpreted as a preliminary mapping of the field rather than as conclusive evidence of the superior effectiveness of any particular methodology.

### Recommendations for future research

This scoping review reveals gaps in studying nocebo effects on motor performance. A priority is refining nocebo induction, particularly with verbal suggestions. Researchers should use standardized scripts while monitoring unintended effects. The timing of suggestions—whether delivered before, during, or after an intervention—should be investigated to determine its impact on the magnitude of the nocebo effect. Combining verbal instructions with conditioning could enhance credibility. Comparative studies are needed to evaluate different induction strategies across administration routes and instructional frameworks.

Experimental designs should also prioritize *between-subject* approaches to minimize carryover effects and isolate properly the effects caused by the nocebo effect. Research should isolate nocebo effects while expanding participant diversity to include older adults, sedentary individuals, and clinical populations like stroke survivors or Parkinson’s patients. This would broaden theoretical insights and inform rehabilitation applications where subtle motor declines matter.

Beyond methodological considerations, current literature lacks sufficient focus on fine motor skills, crucial in daily functioning. Future studies should incorporate precision-based tasks—such as digitized handwriting, or VR-assisted tests—using motion capture. These paradigms could be further enriched by introducing real-world complexity, such as dual-task conditions, to better capture nocebo effects in ecologically valid settings.

Addressing these gaps will improve understanding of nocebo effects on motor performance, informing interventions in sports, rehabilitation, and daily functioning.

## Conclusion

This scoping review examined the methodologies, measurement methods, and observed effects of nocebo interventions on motor performance in healthy individuals across 18 studies. The analysis identified verbal instruction as the most prevalent induction method, particularly when enhanced through conditioning or visual cues. Regarding administration routes, electrophysical and oral interventions successfully showed nocebo effects in their outcomes, while topical and injection methods did not. Sham nocebo treatments were more effective than active nocebo interventions, reinforcing the role of expectation in nocebo responses. This review identified a predominant focus on gross motor tasks (e.g., cycling, sprinting, postural control), with fine motor tasks (e.g., precision gripping, handwriting) remaining underrepresented. Outcome measures predominantly focused on performance declines (e.g., reduced force, endurance) rather than qualitative movement degradation (e.g., coordination, stability). Between-subject designs demonstrated higher sensitivity in detecting nocebo effects compared to within-subject approaches.

Future research should prioritize standardized induction protocols, expanded participant diversity (including clinical populations), and tasks focused on fine motor control.

## Data Availability

The original contributions presented in the study are included in the article/Supplementary material, further inquiries can be directed to the corresponding author.
